# Effects of Chinese pediatric therapeutic massage on the management of functional constipation in children: A randomized single blind trial

**DOI:** 10.1097/MD.0000000000046865

**Published:** 2025-12-26

**Authors:** Xue Bai, Dandan Li, Yukui Tian, Jing Xian Li, Qingguang Zhu, Lingjun Kong, Junchang Liu, Cheng Wang

**Affiliations:** a Department of Acupuncture and Moxibustion and Massage, College of Traditional Chinese Medicine, Xinjiang Medical University, Urumqi, China; b Department of Acupuncture and Tuina, Affiliated Hospital of Traditional Chinese Medicine, Xinjiang Medical University, Urumqi, China; c School of Human Kinetics, Faculty of Health Sciences, University of Ottawa, Ottawa, Canada; d Department of Physical Therapy with Chinese Massage, Yueyang Hospital of Integrated Tradition al Chinese and Western Medicine, Shanghai University of Traditional Chinese Medicine, Shanghai, China; e Department of Massage, Affiliated Shuguang Hospital, Shanghai University of Traditional Chinese Medicine, Shanghai, China.

**Keywords:** children, Chinese manual manipulation therapy, functional constipation, Tuina

## Abstract

**Background:**

Functional constipation (FC) is a common health issue in children, impacting their health-related quality of life. Chinese pediatric therapeutic massage, also called Chinese pediatric Tuina, a traditional Chinese manual manipulation therapy, has been utilized for managing FC in children. This study examined the effects of 2 types of pediatric Tuina therapy, regular and Han’s style, on FC in children diagnosed based on the Rome IV criteria and with the type of food stagnation accompanied by interior heat/inflammation in traditional Chinese medicine.

**Methods:**

The study involved 196 children aged 1 to 7 years (mean [M] age 2.7 ± 1.5 years) diagnosed with FC for 3 to 6 months based on Rome IV criteria and traditional Chinese medicine’s diagnosis of food stagnation with internal heat/inflammation. Two groups of 98 participants each were randomly formed. One group received regular pediatric Tuina therapy, while the other received Han’s style pediatric Tuina therapy for 2 sessions. Each session lasted for 7 days with a one-day break, and a 3-day break was scheduled between the 2 sessions. Bristol stool scale, scale of defecation difficulty, scale of stooling duration, scale of stooling frequency, and abdominal bloating were assessed at pretreatment, post-first session, post-second session, and 1-week after the second session. Paired *t*-tests and independent *t*-tests were used to examine significant differences in measures among data collected from different time points within each group and between the 2 groups, respectively.

**Results:**

Both types of Tuina therapy (regular and Han’s style) demonstrated significant improvements in all measures compared to pre-Tuina (*P* < .05). Han’s style Tuina showed significantly better effects (*P* ≤ .01) than regular Tuina.

**Conclusion:**

Chinese pediatric Tuina therapy significantly improved FC symptoms caused by food stagnation with interior heat/inflammation in the studied population, with Han’s style having certain advantages. The Chinese Tuina therapy used in the study could be recommended as an alternative management approach for FC in children.

## 1. Introduction

Functional constipation (FC)is a common health issue in childhood, prevalence rate was between 0.7% and 29.6% (median 12%).^[1]^ Chinese children ages 6 to 7 years had a notably higher prevalence of 16.8% prevalence.^[2]^ FC significantly impacts the health-related quality of life of affected children and also affects the lives of their parents and family.^[3]^ It is characterized by infrequent, painful, and hard stools and may be accompanied by fecal incontinence and abdominal pain.^[4]^ FC is diagnosed clinically based on history and physical examination and is defined according to the Rome IV criteria.^[5,6]^ According to international guideline,^[7,8]^ the treatment for children with FC includes demystification, education, toilet training, and laxative treatment with polyethylene glycol. However, due to the persistent and challenging nature of FC in children, there has been exploration into complementary or alternation medicine for treatment.^[9,10]^

Tuina, traditional Chinese manual manipulation or therapeutic massage, has been applied for management of functional disorders of gastrointestinal system in both adults and children. It is among the most commonly employed modalities in the noninvasive therapeutic practices of traditional Chinese medicine and has been practiced for thousands of years in China, now extending globally.^[11]^ Pediatric Tuina is a specialized branch of Tuina therapy focused on addressing health issues in babies, toddlers and children. Pediatric Tuina involves the use of specific acupoints located mainly on the fingers, palms, arms, head, abdomen, and back of children. Additionally, pediatric Tuina targets the manipulation of the meridian pathways in superficial layer of the body. A distinguishing characteristic of pediatric Tuina is its use of faster and gentler manipulation motions compared to Tuina therapy for adults.^[12]^ The effectiveness, safety, and practicality of pediatric Tuina have been documented.^[13]^ Over the course of thousands of years of practice, pediatric Tuina has developed general guidelines for managing FC in children.^[14]^ Furthermore, unique styles of pediatric Tuina have emerged, for example Han’s style pediatric Tuina. It is originated from “One Finger Zen Tuina” and is characterized by manipulation of meridians, acupoints, or specific body positions with precise pressure, rapid yet gentle movements, and a focus on avoiding stagnation. The manipulation motions in Han’s style Tuina can reach speeds of up to 200 times/min.

The effects of pediatric Tuina on the management of FC in children have been studied. Two research teams published meta-analyses regarding the application of Tuina in treating FC in children.^[15,16]^ Both studies analyzed a substantial number of published original studies, with 1 including 16 studies comprising 1424 cases, and the other involving 23 randomized controlled trials totaling 2005 cases. Their findings indicated that Tuina therapy significantly improved fecal characteristics, such as increasing defecation frequency. Furthermore, Tuina therapy, being a non-pharmacological and noninvasive approach, has demonstrated good acceptance among children. The aforementioned studies suggest the need for further research with high quality and larger sample sizes to provide additional scientific evidence regarding the efficacy of Tuina therapy in managing FC in children.

The aim of this study was to examine the impact of pediatric Tuina therapy on FC caused by food stagnation with interior heat/inflammation in children. Additionally, besides regular pediatric Tuina therapy, the effects of Han’s style pediatric Tuina therapy on FC in children were also examined. It was expected that the findings from the study could contribute to a better understanding of Han’s style pediatric Tuina therapy in managing FC in children. Hopefully, the results could provide scientific evidence in the effects of Tuina therapy specifically on FC caused by food stagnation with interior heat/inflammation in children.

## 2. Materials and methods

### 2.1. Participants

This study protocol was reviewed and approved by the Research Board of Human Ethics at the Hospital Authority, Xinjiang Medical University Studies (approval number: 2023XE0177-1). Written informed consent was obtained from participant’s parent to participate in the study in compliance with the Helsinki Declaration. A total of 196 children aged 1 to 7 years (M age 2.7 ± 1.5 years) participated in the study. The participants were recruited through the Department of Acupuncture and Tuina at the Hospital of Traditional Chinese Medicine affiliated with Xinjiang Medical University. The inclusion criteria were as follows: diagnosed with FC for 3 to 6 months according to Rome IV criteria; 2) diagnosed with the constipation type of food stagnation with internal heat according to the diagnostic criteria of traditional Chinese medicine. This type of FC in children is characterized by halitosis, a red-colored dorsal surface of the tongue with a thick yellow coating, warmth in the palms and soles, a strong wrist pulse, and purplish stagnation of the superficial surface veins in interphalangeal digital creases in children younger than 3 years old;^[17]^ 3) did not receive medical intervention in the last 3 months; did not accept any other interventions, such as changing diet or taking medication or nonprescription products for constipation; parents or legal guardians were seeking alternative medicine therapy and were motivated to participate in the study; without any other medical conditions except for FC; and presented normal growth and development as well as cognitive function. The exclusion criteria were as follows: children under 3 months or over 6 months old; constipation caused by organic pathologies (confirmed through imaging studies and laboratory screening); children with concurrent life-threatening conditions or neuropsychiatric disorders; children presenting contraindications to Tuina therapy, including dermatological diseases or skin lesions and children unwilling to cooperate with the treatment protocol.

### 2.2. Tuina therapy

Eligible participants were randomly assigned into 2 groups of 98 participants each. One group received the regular pediatric Tuina that is commonly applied in pediatric Tuina practice.^[18,19]^ The other group received Han’s style pediatric Tuina that applies unique acupoints/positions of the body and Tuina techniques. The selected acupoints, associated meridians, and positions for Tuina in both groups are presented in Table 1.

**Table 1:** Acupoints/positions and associated meridians utilized in both groups.

Acupoints/position	Associated meridians	Regular Tuina	Han’s style Tuina
*Bo Yang Chi (L11	Large intestine meridian (L) (Shouyangming dachangjing)	+	+
* Zu San Li (ST36)	Stomach meridian (ST) (Zuyangming xiaochangjing)	+	−
*Qi Jie Gu (GB30)	Gallbladder meridian (GB) (Zushaoyang danjing)	+	+
*Gui Wei/Chang Qiang (GV1)	Governor vessel (GV) (Du mai)	−	+
*Lao Gong Xue (PC8)	Pericardium meridian (PC) (shoujueyin xinbaojing)	−	+
*Tian Shu Xue (ST 25)	ST	−	+
Abdomen	Distribution site of ST, spleen meridian (SP), conception vessel (CV)	+	+
Lateral side of thorax	Distribution site of liver meridian (LR) & GB	+	−
Spine	Distributed site of GV	+	−
Later side of index finger	Distribution site of L	−	+
The palmar creases of second to fifth fingers	Related to function of digestive system	−	+

*, acupoint; “+,” applied; “−”, wasn’t applied; (), the international codes for meridians and collaterals.^[19]^

**Figure 1.**
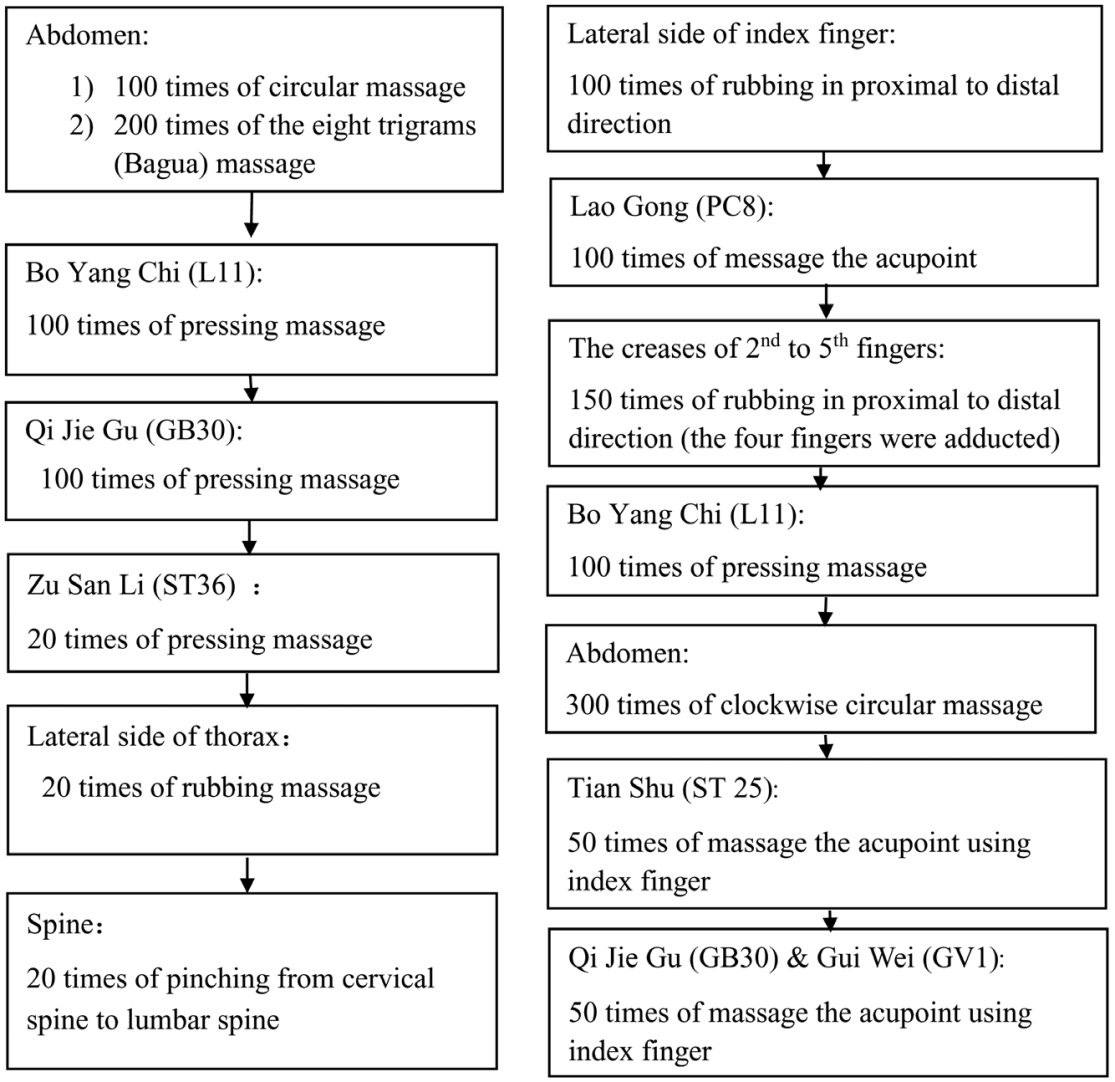
Manipulation acupoints/position, techniques, and sequence of regular pediatric Tuina (left column) and Han’s style pediatric Tuina (right column) in the study.

The participants received either regular pediatric Tuina or Han’s style pediatric Tuina for 2 sessions. Each session lasted for 7 days with a 1-day break after the third day of treatment. A 3-day break was scheduled between the 2 Tuina sessions. The manipulation protocols for regular pediatric Tuina and Han’s style pediatric Tuina are presented in Figure 1. All Tuina therapy sessions were conducted by specialized Tuina doctors.

### 2.3. Measures and statistical analysis

The parents of the participants were instructed to maintain a stool diary for their child, which included information such as stool frequency, duration of each stooling episode, and stool form. A mobile app for recording the stool diary was installed on their mobile phones, and printed diary forms were also provided as an alternative option. Stool diary data were collected at 3 time points: pre- and post-first session of Tuina therapy, post-second session of Tuina therapy, and 1-week after the second Tuina therapy session. The data collection was managed by the researchers, who were pediatric Tuina doctors. Please refer to the guidelines flow diagram for details.

The outcome measures included:

1. Bristol stool scale.
2. Scale of defecation difficult rated on a scale of 1 to 3, with 1 for easy, 2 for somewhat hard, and 3 for vary hard.
3. Scale of stooling duration represented by ratings from 0 to 3, with 0 for stooling duration shorter than 10 minutes, 1 for stooling duration between 10 and 15 minutes, 2 for 16 and 25 minutes, and 3 for longer than 25 minutes.
4. Scale of stooling frequency rated on a scale of 1 to 3, with 1 for 3 times/wk, 2 for 2 times/wk, and 3 for 1 time/wk.
5. The scale of abdominal distension is rated on a scale of 1 to 3. Among the following 4 symptoms: swollen abdomen, resistance or tension felt during palpation, tympanic sounds upon percussion, and decreased or absent bowel sounds, a score of 1, 2, and 3 represents the presence of 1, 2, or 3 of the symptoms, respectively.

The statistical analysis of the data was conducted using SPSS version 23.0 (Armonk). Analysis results were presented as M and standard deviation. A paired *t*-test was applied to assess significant differences in the measures among the data collected from 3 time points within each group, respectively. An independent *t*-test was used to examine significant differences in the measures between the 2 groups at each data collection time point. Statistical significance was set at *P* ≤ .05.

The efficacy of pediatric Tuina therapy for FC was evaluated based on the criteria for effective evaluation in traditional Chinese medicine treatment,^[20]^ which consisted of 3 categories: success, improvement, and slight improvement. Category 1 referred to children who had stools with a Bristol scale type of 3 to 5 once a day after completing Tuina therapy. Category 2 included children who had stools twice a week, with stools becoming softer than before therapy. “slight improvement” referred to those with no improvement in symptoms after Tuina therapy. The efficacy rate was expressed as a percentage, calculated using the formula: (total cases − cases of slight improvement)/total cases × 100%. A chi-square test (*χ*^2^) was used to examine the significance of Tuina therapy effectiveness between pre-Tuina therapy and post-Tuina therapy in both groups. The significance level was set at *P* < .05.

## 3. Results

Table 2 displays the M and standard deviation of the measures obtained from each measuring time point in both groups. Compared to the measures obtained pre-Tuina therapy, the Bristol scale scores significantly increased after the first session, second session, and 1-week after 2 sessions of Tuina therapy in both groups (*P* < .05). The scale of defecation difficulty significantly decreased in both groups, indicating easier stooling compared to pre-Tuina therapy. Stooling duration was significantly shorter in both groups compared to pre-Tuina therapy measures. Stooling frequency also significantly increased after Tuina therapy.

**Table 2:** Mean (M) and standard deviation (SD) of the measures in both groups at different evaluation times.

	Regular pediatric Tuina group				Han’s style pediatric Tuina group			
Measures	Pre-Tuina therapy	Post-first session	Post-second session	1-week post second session	Pre-Tuina therapy	Post-first session	Post-second session	1-week post second session
Bristal stool scale	1.61 ± 0.57	1.85 ± 0.44	2.12 ± 0.33	2.23 ± 0.43	1.64 ± 0.63	2.03 ± 0.17	2.37 ± 0.51	2.82 ± 0.40
Difficult defecation scale	2.20 ± 0.42	2.12 ± 0.33	2.03 ± 0.17	1.68 ± 0.51	2.18 ± 0.41	2.01 ± 0.10	1.85 ± 0.42	1.55 ± 0.54
Stooling duration scale	2.38 ± 0.55	2.28 ± 0.45	2.14 ± 0.41	2.01 ± 0.10	2.42 ± 0.52	2.14 ± 0.43	2.01 ± 0.18	1.94 ± 0.24
Stooling frequency scale	2.32 ± 0.51	2.27 ± 0.44	2.21 ± 0.50	2.03 ± 0.17	2.37 ± 0.53	2.13 ± 0.34	2.10 ± 0.30	1.94 ± 0.24
Abdominal distension	2.55 ± 0.54	2.37 ± 0.51	2.22 ± 0.44	2.07 ± 0.33	2.74 ± 0.48	2.28 ± 0.45	2.16 ± 0.42	1.96 ± 2.25

M = mean, SD = standard deviation.

The results of the chi-square test assessing the efficacy of Tuina therapy in both groups are presented in Table 3. Both regular pediatric Tuina therapy and Han’s style pediatric Tuina therapy demonstrated significantly high efficacy after treatment (*P* < .05). The total effective rate reached 79% in the regular pediatric Tuina therapy group and 91% in the group that received Han’s style pediatric Tuina therapy.

**Table 3:** Cases of success, improvement, and slight improvement as well as their efficacy rate (%) after the Tuina therapy in 2 groups and statistical analysis result.

Group	Success	Improvement	Slight improvement	Total effective cases
Regular pediatric Tuina therapy (n = 98)	53 (54.08)	26 (26.53)	19 (19.39)	79 (80.61)
Han’s style pediatric Tuina therapy (n = 98)	67 (68.37)	24 (24.49)	7 (7.14)	91 (92.86)
*Z* value or *χ*^2^ value	*Z* = 2.391			*χ*^2^ = 6.386
*P* value	.017			.012

## 4. Discussion

This study investigated the impact of pediatric Tuina therapy on the management of FC in children. The findings indicated that 2 weeks of Tuina therapy, including both regular pediatric Tuina and Han’s style pediatric Tuina, significantly relieved FC symptoms, in terms of stool characteristics, stool frequency, stooling difficulty, and stooling duration, with an efficacy rate exceeding 80%. Notably, Han’s style pediatric Tuina demonstrated higher efficacy compared to the regular approach.

In traditional Chinese medicine, FC in children can arise from functional disorders of the gastrointestinal system.^[17]^ It falls under the category of “constipation disease,” characterized by “difficulty in defecation” or “difficult bowel movements.” The etiology of this condition can be attributed to 2 main pathogenic factors: external environmental changes and internal environment imbalance. External factors include alterations in living environments such as extreme temperatures (heat or cold), humidity levels (very high or low), and the presence of pathogenic bacteria. Internal factors encompass disturbances in the body’s internal environment balance or hemostasis, which can be linked to diet, digestive function, or abnormal bowel movements. Treatment principles for FC aim to promote digestive function, relieve stagnation, and regulate the functions of organs involved in digestion, absorption, and bowel movement. Studies have demonstrated that traditional Chinese Tuina therapy is effective in treating FC in children and have become a regular treatment approach,^[15,18]^ offering the advantages of non-pharmacological treatment, convenience, and cost-effectiveness. The results of this study align with previous findings, highlighting that pediatric Tuina therapy, whether regular or Han’s style, significantly alleviates FC symptoms.

Han’s style pediatric Tuina is influenced by One Finger Zen Tuina. It specifically targets the promotion of gastrointestinal peristalsis and the adjustment of gastrointestinal microenvironment hemostasis. This style encompasses not only the techniques of regular pediatric Tuina but also introduces unique methods such as reverse moving of the inner 8 trigrams and manipulation of specific positions, including the palmar creases of the second to fifth fingers, the lateral side of the index finger, and the acupoints Qi Jie Gu and Gui Wei. These acupoints and positions correspond to certain meridians associated with gastrointestinal function and internal environment hemostasis. Manipulating these targeted areas is believed to enhance gastrointestinal function, remove excess heat from the body, and restore internal balance.^[21]^ The techniques employed in Han’s style pediatric Tuina, such as kneading and shaking as used in the study, are characterized by faster yet gentle motions with the appropriate pressure or mechanical forces compared to regular pediatric Tuina techniques. These techniques may influence gastrointestinal motility, promote peristalsis, and thereby facilitate bowel movements.^[22,23]^ The combination of Han’s style pediatric Tuina techniques and the application of specific acupoints/positions in the study likely contribute to its higher efficacy rate in managing FC in children.

The effects of the 2 styles of pediatric Tuina therapy on FC in children are closely linked to their targeted acupoints/positions and manipulation techniques. Research in acupuncture and neuromodulation has provided evidence of acupoints’ responses to mechanical stimulation from needling. Takahashi reviewed literature published since 1975 regarding the mechanisms of acupuncture’s neuromodulation in the gut.^[24]^ Based on these studies, Takahashi summarized that acupuncture stimulates the somatic afferent nerves of the skin and muscles. The somatic sensory information from the body is carried to the cortex area of the brain. Somatic sensory fibers also project to the various nuclei at the brain stem and hypothalamus. Via somato-autonomic reflex, acupuncture modulates various biomechanical responses, such as prokinetic, antiemetic, and antinociceptive effects. Tuina manipulation applies mechanical forces with specific magnitudes, frequencies, durations, and force development rates to the targeted acupoints/positions. This manipulation serves as mechanical stimulation to the applied acupoint/position, akin to acupuncture. Therefore, Tuina therapy may produce similar stimulation effects to acupuncture, contributing to its efficacy in treating FC in children.

Traditional Chinese medicine posits the existence of channels known as meridians that connect the body’s surface to internal organs. Langevin and Yandow conducted a study on the relationship between acupuncture points and meridians with connective tissue planes.^[25]^ They proposed that the network of acupuncture points and meridians could be considered a representation of the network formed by interstitial connective tissue. Using ultrasound images demonstrating connective tissue cleavage planes at acupuncture points in normal human subjects, they observed an 80% correspondence between acupuncture point sites and the location of intermuscular or intramuscular connective tissue planes in postmortem tissue sections. They suggested that the anatomical relationship of acupuncture points and meridians to connective tissue planes is pertinent to understanding acupuncture’s mechanism of action. From a Western medicine perspective, the aforementioned study’s evidence may elucidate why manipulation of target acupoints/positions affects symptoms of FC in children. This is because manipulation of acupoints/positions on the skin may impact the connective tissues within the gastrointestinal system. This connection between acupuncture points and connective tissue planes could contribute to the understanding the therapeutic effects observed in Tuina therapy for FC.

This study still has the following limitations. First, the trial had a small sample size and may be defined as an exploratory study. Second, the research only focused on the efficacy of the short-term (1-week post-second session), leaving the long-term effect still to be elucidated. Statistical analysis could be improved with the method of per-protocol analysis and the method of using multiple imputation method for missing data processing. At the same time, sensitivity analysis can further increase the accuracy and guidance of the results. Therefore, based on the results of this trial, future work should expand the sample size, increase the observation period and follow-up period, more refined statistical methods, in order to achieve higher clinical significance.

## 5. Conclusion

Chinese pediatric Tuina therapy has significant benefits in managing FC caused by food stagnation with interior heat/inflammation in children aged 1 to 6 years old who have had the condition for 3 to 6 months. Both regular pediatric Tuina and Han’s style pediatric Tuina showed significant therapeutic effects, with Han’s style pediatric Tuina therapy having certain advantages. The Tuina approaches used in the study indicate that food stagnation with interior heat/inflammation could be effectively regulated by Tuina. Therefore, pediatric therapeutic Tuina can be recommended as an alternative management approach for FC in children.

## Acknowledgments

Authors declare that to thank all participants for acceptance to join the trial.

## Author contributions

**Data curation:** Xue Bai, Dandan Li.

**Formal analysis:** Jing Xian Li, Qingguang Zhu.

**Funding acquisition:** Dandan Li, Cheng Wang.

**Investigation:** Yukui Tian.

**Methodology:** Jing Xian Li.

**Software:** Yukui Tian.

**Supervision:** Lingjun Kong.

**Writing – original draft:** Xue Bai.

**Writing – review & editing:** Junchang Liu.
